# Understanding Is Key: An Analysis of Factors Pertaining to Trust in a Real-World Automation System

**DOI:** 10.1177/0018720818761256

**Published:** 2018-04-03

**Authors:** Nora Balfe, Sarah Sharples, John R. Wilson

**Affiliations:** Trinity College Dublin, Ireland; University of Nottingham, UK

**Keywords:** human-automation interaction, supervisory control, technology acceptance, trust in automation, ethnographic observations

## Abstract

**Objective:**

This paper aims to explore the role of factors pertaining to trust in real-world automation systems through the application of observational methods in a case study from the railway sector.

**Background:**

Trust in automation is widely acknowledged as an important mediator of automation use, but the majority of the research on automation trust is based on laboratory work. In contrast, this work explored trust in a real-world setting.

**Method:**

Experienced rail operators in four signaling centers were observed for 90 min, and their activities were coded into five mutually exclusive categories. Their observed activities were analyzed in relation to their reported trust levels, collected via a questionnaire.

**Results:**

The results showed clear differences in activity, even when circumstances on the workstations were very similar, and significant differences in some trust dimensions were found between groups exhibiting different levels of intervention and time not involved with signaling.

**Conclusion:**

Although the empirical, lab-based studies in the literature have consistently found that reliability and competence of the automation are the most important aspects of trust development, understanding of the automation emerged as the strongest dimension in this study. The implications are that development and maintenance of trust in real-world, safety-critical automation systems may be distinct from artificial laboratory automation.

**Application:**

The findings have important implications for emerging automation concepts in diverse industries including highly automated vehicles and Internet of things.

## Introduction

Automation is becoming increasingly pervasive, and there is an urgent need to learn lessons from well-established highly automated systems in order to influence the design of new forms and applications of automation. This paper explores the role of factors pertaining to trust in real-world automation systems, applying observational methods in a case study from the railway sector to relate automation use to reported trust. The system under investigation is an advanced automated control system that has been successfully operating on portions of the UK rail system for several decades. Trust has been identified as a potentially important construct in relation to automation by researchers who theorize that low levels of trust in automation may influence operators’ usage (e.g., Muir, 1987; Sheridan, 1999). Muir (1994) stated that if it was not possible to build automated systems that are trustworthy, then we could not build automated systems at all, and several studies have found a correlation between trust levels and use of automated systems (e.g., de-Vries, Midden, & Bouwhuis, 2003; Lewandowsky, Mundy, & Tan, 2000; Moray, Inagaki, & Itoh, 2000; Muir & Moray, 1989). Typically these studies find that operators use automation only to the extent that they trust it; if operators distrust automation they will reject it, preferring to perform the task manually. However, almost all these experiments were laboratory based, utilizing simulations of work environments and whether these results transfer to real work environments is still an open question—but an important one given the increasing levels of automation in the workplace.

The work of both Barber (1983) and Rempel, Holmes, and Zanna (1985) forms the most common basis for a definition of trust in relation to human-automation interaction. These definitions were originally developed to represent interpersonal trust but have been commonly adapted to define human-automation trust (Madhavan & Wiegmann, 2007). Both are three-stage definitions and can be regarded as somewhat overlapping. The first stage involves the creation of an accurate mental model that allows the operator to understand and predict the behavior of the system (Muir, 1987). This implies that trust is dependent upon understanding of the system (Lee, 1991). The second stage concerns the ability of the system to correctly perform its tasks and is frequently regarded as the most important for human-automation trust (Muir, 1987). This might also be called *reliability* or *competence* and refers to the performance of the system (Lee, 1991). Barber identified three types of technical competence that one human might expect from another: expert knowledge, technical facility, and everyday routine performance. Automation may be capable of carrying out only one of these three factors but still be able to perform its individual task satisfactorily. The final dimension can be labeled *faith* and becomes important when the automation is more competent than the human operator. The operator is therefore unable to evaluate the automation and must rely on an assessment of the automation’s responsibility. Several researchers have examined the factors influencing trust, and these are summarized in two studies (Hoff and Bashir, 2015; Schaefer et al., 2014). Both suggest that trust is influenced by three sources: The first is related to the individual, including his or her personality, background, attitudes, and capabilities, and can be labeled *dispositional trust.* The second relates to the situation or environment, including the complexity of the task and the operator workload, and can be labeled *situational trust.* The third relates to the automation itself, including its behavior, reliability, transparency, and performance, and can be labeled *learned trust.*

The foregoing definitions suggest that trust is a multidimensional concept and that there are many factors that can influence an operator’s trust in an automated system. Research, much of it using a pasteurization plant simulation, has consistently shown that automation reliability is closely related to operator trust (Lee & Moray, 1994; Muir & Moray, 1989; Wiegmann, Rich, & Zhang, 2001). In fact, there are two facets to reliability; an automated system may be reliable in the sense that it does not suffer mechanical failure, but it must also be reliable in the sense of making correct decisions consistently or performing its function well. This second facet can be labeled “competence” for clarity (Madsen & Gregor, 2000; Muir & Moray, 1996; Parasuraman, Sheridan, & Wickens, 2000). System competence has been found to be the greatest predictor of the operator’s overall trust (Muir & Moray, 1989), and operator trust may be affected differently by different levels of system incompetence. Small errors, even those that do not affect performance, may greatly reduce trust, whereas operators have been found to become increasingly less sensitive to larger errors (Lee & Moray, 1992; Muir & Moray, 1996). Automation must therefore be extremely reliable if high levels of trust and usage are to be achieved; Wickens and Dixon (2007), in a meta-analysis, found that less than 70% reliability of automation is worse than useless.

Such research highlights the importance of highly reliable and competent automation; however, operators may perceive even unreliable automation to be better than manual operation. Riley (1996) suggested that operators’ trust in, or decision to rely on, automation is strongly influenced by the operators’ self-confidence. If an operator has more confidence in his or her own abilities than in the automation, then he or she is more likely to perform the task manually, and research using the pasteurization plant simulation has confirmed this relationship (Lee & Moray, 1992, 1994). Participants used the automated system when conditions became such that they could not manage the system manually (e.g., during faults). Despite the low reliability, the automation became useful to the operator (Sheridan, 1999). The type of automation error and the consequences of that error also influence usage (Jiang et al., 2004); for example, if the automation makes an incorrect decision that causes further problems for an already overloaded operator, the operator is more likely to discontinue using the automation. The interplay between competence, usefulness, and self-confidence may be quite complex, but to ensure automation is useful and utilized, the literature is clear that the first requirement is reliability, both in the sense of repeated consistent functioning and competent decision making.

Safety-critical systems are likely to be highly reliable and competent, and operators highly trained and confident in their abilities, and in these cases other factors may influence trust. Feedback from the automation, and its transparency, becomes particularly important as automation becomes more complex and possibly even exceeds operator competence. Operators require explicit and appropriate feedback about its intentions in order to develop appropriate expectations (Sarter, Woods, & Billings, 1997; Sheridan, 1999). Good feedback may even counter the loss of trust in automation with low reliability and increase automation use. Research has shown that if operators are given an explanation as to why the automation might err then trust and usage levels can be maintained (Beck, Dzindolet, & Pierce, 2007; Dzindolet, Peterson, Pomranky, Pierce, & Beck, 2003). Simpler automation systems may not require advanced levels of feedback as the operator may be capable of understanding and predicting the automation without such prompts. It is the ability to develop an accurate mental model (see Wilson & Rutherford, 1989, for a description of mental models) that the operator can use to understand and accurately predict future behavior of the system that facilitates trust (Madsen & Gregor, 2000; Muir & Moray, 1996; Sheridan, 1999). Wickens (1992) stated that successful performance in control rooms depends on a good mental model of the system, allowing operators to anticipate future system states, formulate plans, and troubleshoot effectively, and poor or inaccurate mental models have been associated with incidents and accidents (Sheridan & Parasuraman, 2006). Operators who possess accurate mental models can make correct judgments about when an automated system can be relied on and when it should not be relied on. This is referred to as *trust calibration.*

*Calibration of trust* refers to the correspondence between a person’s trust in the automation and the automation capabilities (Lee & See, 2004; Madhavan & Wiegmann, 2007). If trust is miscalibrated, the result is inappropriate reliance on the automation, either overtrust or undertrust. For a system to work optimally, the operator’s level of trust in the automation must be correctly calibrated (i.e., it should match the actual capabilities of the automation). These capabilities may vary in different circumstances; for example, automation may be competent in one set of circumstances but not in another. Operators should be able to recognize when automation can be relied upon and when it cannot. However, trust is not always uniform between different operators. Preexisting factors such as experience with technology and familiarity may influence operator trust (Sheridan, 1999), meaning that individuals may have different trust levels for the same automated system. Merritt and Ilgen (2008) found that individual differences did affect perceptions of automation competence and hence influenced trust. Interestingly, they also found that individuals who had higher expectations of and a propensity to trust automation had the largest negative impact on trust when the automation failed. This suggests that correct calibration, and optimal automation usage, for less than perfectly reliable automation may more likely be achieved by individuals who are not predisposed to trust the automation.

The vast majority of the research on trust between humans and automated systems has been conducted in lab-based studies, with comparatively little known about how the research findings transfer to real systems. Hoffman, Johnson, and Bradshaw (2013) highlight that much of the research into trust between humans and automation has involved small-world studies using simulation and college students as participants, and they caution that these results do not necessarily generalize. There exist some fundamental reasons why lab findings may not be representative of actual operations. For example, lab studies use novice participants who have limited understanding of the domain of application and limited exposure to the specific system under study. Some aspects of trust may take time to develop—time that is not available in lab experiments. Incentives for participants in lab studies are necessarily a proxy for real-world incentives and may result in different behaviors. Lab studies also typically examine the use of automation as either all or nothing, on or off. This is possibly due to the nature of the automated systems used in the experiments, which do not allow participants to simply intervene to force a decision; however, this approach is possible with some real-world automation systems, including that studied in this work. Some real-world studies do exist, such as that reported by Lyons, Ho, et al. (2016) into test pilot trust in a prototype collision avoidance system. This qualitative study found that the false alarm rate, system performance, transparency, and familiarity with the flight maneuver all influenced pilots’ trust in the system. However, the degree to which this influences their interactions with the automation was not studied. The study reported in this paper aimed to investigate the relationship between trust and automation use in a real-world railway-signaling system through the measurement of reported trust via a questionnaire and observations of automation usage.

Trust is a multidimensional concept and is difficult to measure as it is dependent on circumstances, features of the automation, and individual differences. There is no direct objective measurement of trust, so measurement tends to depend on subjective ratings on the dimensions believed to influence trust, including reliability, competence, understandability, faith, personal attachment, and deception (Atoyan, Duquet, & Robert, 2006; Bisantz & Seong, 2001; Jian, Bisantz, & Drury, 2000; Madsen & Gregor, 2000). Reliability and competence of the automation are known to be fundamental requirements in the development of trust (Muir & Moray, 1989; Wiegmann et al., 2001), but as signaling systems are safety critical, the automation is required to be highly reliable. It may be expected, therefore, that other dimensions in the development of trust may emerge, some of which may not have as strong an empirical basis in the research. These include feedback (Sarter et al., 1997), understandability and predictability (Sheridan, 2002), and faith (Muir, 1987).

The following key dimensions were identified from the literature to be included in the measurement of factors pertaining to trust:

- reliability—in terms of both mechanical reliability and consistent functioning over time (Madsen & Gregor, 2000; Muir & Moray, 1996; Sheridan, 1999);
- robustness—the ability to function under a variety of circumstances (Sheridan, 1999; Woods, 1996);
- understandability—the ability to understand what the automation is doing, why it is doing it, and how it is doing it (Madsen & Gregor, 2000; Sheridan, 1999);
- competence—the perceived ability of the automation to perform its tasks (Madsen & Gregor, 2000; Muir & Moray, 1996);
- feedback—the ability of the automation to explicitly give feedback on its intended actions (Norman, 1990; Sheridan, 1999);
- dependability—the extent to which the automation can be counted on to do its job (Muir & Moray, 1996; Rempel et al., 1985);
- personal attachment—the extent to which operators like to use the automation (Madsen & Gregor, 2000);
- predictability—the ability of the operator to predict the actions of the automation (Muir, 1994; Rempel et al., 1985); and
- faith—the extent of belief that the automation will be able to cope with future system states that it may not yet have encountered (Madsen & Gregor, 2000; Muir, 1994; Rempel et al., 1985).

## Overview of Case Study

Railway signaling control is a critical aspect of railway operations, with responsibility for safely and efficiently implementing the paths of trains through the network. Signaling systems have evolved from direct manual control of small sections of the railway developed in the 1800s to computerized systems featuring advanced automation in the past couple of decades. See Balfe, Wilson, Sharples, and Clarke (2007) for more information about the different generations of signaling systems and a description of the varying levels of automation present. The research presented in this paper examines individual differences in trust when using the Automated Route Setting (ARS) system.

ARS is a fully automated signaling control system, capable of running all timetabled train services under normal operations. It uses timetable information to set routes for trains and incorporates algorithms to make decisions about the prioritization of trains running late. It essentially operates as an invisible operator, setting routes for trains as they traverse the workstation. See Balfe, Sharples, & Wilson (2015) for more details on ARS functionality. The human operators (signalers) are responsible for monitoring the automation and ensuring that the correct routes are set for trains at the correct time and in the correct order. Their aim is to minimize delay, and they also may have some tasks to complete that are outside ARS’s capabilities, but these are relatively few during normal operations. ARS does not give an indication of which route it will set, so signalers rely on their experience and knowledge of the system to choose when to intervene. They can choose to fully disable the automation, but they also have the option to work alongside the automation by disabling the automation over a particular section of the track and/or setting routes before it has the opportunity. Previous research (Balfe, Wilson, Sharples, & Clarke, 2012) has established that turning off the automation entirely is relatively rare and generally occurs only during extreme disruption, but interventions to constrain or force decisions are common. Thus, the automation and the signalers usually work in parallel, with ARS’s setting the majority of the routes and the operators’ monitoring the automation and making brief interventions when they deem it necessary. These interventions therefore represent a disuse of the automation by the signaler.

The aim of the study was to develop and apply a methodology for observing signalers at their workstations and to relate the observed activities to reported levels of trust. This methodology was applied in pursuit of the following objectives:

1. to determine what proportion of signalers’ time is spent monitoring, controlling, planning, communicating, or not actively involved in signaling during normal operations;
2. to establish whether factors pertaining to trust are related to how often the signaler intervenes and their level of monitoring; and
3. to investigate whether the effects between trust and automation usage found in laboratory studies and documented in the literature are applicable with real-world automation systems.

## Method

The research applied an observational method to collect data on signaler activity (Method 1), and trust was measured via a questionnaire (Method 2). This research complied with the University of Nottingham’s ethics code and was approved by the ethical review board at the university. Informed consent was obtained from each participant.

### Observational Method

A coding scheme was developed to support the manual real time observations in the field. Five basic codes were used:

- monitoring,
- intervening,
- planning,
- communicating, and
- quiet time.

A sixth supplementary code (closed-circuit television [CCTV]) was added for one of the signal boxes included in the study. It is important to note that monitoring was coded when it was the only activity in which the signaler was engaged. Within these five categories, additional subcategories were coded. These are described in Table 1. We might expect that signalers with lower trust would engage in higher levels of monitoring in anticipation of having to intervene, higher intervention levels, and lower levels of quiet time as they do not trust the automation to continue unsupervised.

**Table 1:** Observation Coding Scheme

Main Code	Subcode	Description
Monitoring	Active monitoring	Monitoring was coded as active if the signaler was sitting up while monitoring.
	Passive monitoring	Monitoring was coded as passive if the signaler was sitting back while monitoring.
Intervention	Trackerball	Trackerball usage was noted only if the signaler used the button (i.e., simply moving the cursor with no resulting intervention was coded as active monitoring).
	Keyboard	Use of the keyboard attached to the automation system only was classified under this heading. Other systems on the workstation also had keyboards, but use of these was classified as planning behavior.
Planning	Planning	Any reference to planning tools included paper versions of the timetable for the area and computer systems showing current train delays in the area.
Communications	Telephone	Any telephone or intercom calls were classified under this heading.
	Voice comms	Communications with the signaler on an adjacent workstation or the shift manager were classified under this heading. Only information that was relevant to the immediate signaling situation was thus classified. Conversations regarding, for example, situations that occurred in the past were coded as *quiet time* as they would not have been relevant to the signaling at that time.
Quiet time	Quiet time	This included any time when the signaler was involved in an activity not directly related to signaling. Conversations with other signalers or staff, conversations with the researcher, and reading newspapers or magazines were all examples of activities classed as quiet time.
	Signaler away from workstation	Signalers occasionally took time away from the workstation, for a variety of reasons but most commonly to make a cup of tea. If the workstation was left unattended this activity was classed as quiet time away from the workstation.
CCTV	CCTV	Only one of the sites had CCTV screens on the workstations. These screens are used to monitor and operate level crossings. When the signaler was involved in either monitoring or operating these, CCTV was coded.

*Note.* CCTV = closed-circuit television.

The coding scheme was tested in a pilot study to ensure that the codes were exhaustive and that it was possible to easily differentiate between the behaviors. The basic coding scheme worked well during the pilot, but a major finding was the existence of different levels of monitoring behavior. The signaler’s position was observed to change substantially during the monitoring task, and during the pilot study five levels were identified. The highest level had the signaler sitting up and watching the screens intently with his or her hand on the trackerball and was very common when the signaler was waiting for the right moment to intervene. In the next level the signaler again was sitting up with his or her hand still on the trackerball but scanning the screens rather than watching one spot intently. This monitoring appeared to be associated with situations where the signaler felt it was likely he or she may have to intervene but had not yet decided where. The next level was similar, but the signaler did not have his or her hand on the trackerball. This was inferred to be pure information gathering monitoring behavior. It was often seen when the signaler was preparing to leave the desk, had just returned, or immediately after an intervention. The next type of monitoring behavior identified was passive monitoring; the signaler was sitting back, but it was clear from his or her movements and posture that he or she was watching what was happening on the screens. The final type of monitoring behavior seen during the pilot was complete passive monitoring. Often the signaler put his or her hands behind the head, and it was not possible to tell if he or she was even focusing on the screens. Although these five levels were clearly observed during the pilot study, to attempt to code these for all observed signalers subsequently would have greatly complicated the observer’s task. Therefore, a decision was made to differentiate between only active and passive monitoring in subsequent observations. Pan, Gillies, and Slater (2008) stated that body movement is an easily observable indicator of a person’s state. Cues in the posture and behavior of signalers were used to infer whether they were engaging in active or passive monitoring.

The interrater reliability of the method was tested and, after second researcher training and familiarization, was found to generate an 80% proportion of agreement and a Cohen’s Kappa of 65%, representing good agreement.

### Questionnaire Method

A questionnaire was administered to gather data on factors pertaining to signalers’ trust in the automation. Statements for each of the key dimensions identified in the literature were taken from previously validated questionnaires (Bisantz & Seong, 2001; Jian et al., 2000; Madsen & Gregor, 2000; Muir & Moray, 1996), and a five-point Likert-type scale (from *strongly agree* to *strongly disagree*) was applied to each statement. The statements were slightly modified to suit the signaling environment, and a final statement—“I trust ARS”—was included to gauge overall trust in the system. The 19 statements on the questionnaire are shown in Table 2.

**Table 2:** Questionnaire Statements

1. ARS is always available for use (Mechanical Reliability).
2. ARS is capable of performing under a variety of different circumstances (Robustness).
3. It is easy to understand what ARS does (Understandability 1).
4. ARS is capable of signaling trains as competently as a signaler (Competence 1).
5. ARS gives explicit information on its intended actions (Feedback).
6. I can count on ARS to do its job (Dependability).
7. I have a personal preference for using ARS (Personal Attachment).
8. I can predict what ARS will do from moment to moment (Predictability 1).
9. If ARS makes a routing decision which I am uncertain about I have confidence that ARS is correct (Faith 1).
10. I understand how ARS works (Understandability 2).
11. ARS performs well under normal running conditions (Competence 2).
12. ARS is very unpredictable, I never know what it is going to do (Predictability 2).
13. I can rely on ARS to function as it is supposed to (Reliability 2).
14. Even if I have no reason to expect that ARS will be able to deal with a situation, I still feel certain that it will (Faith 2).
15. I understand why ARS makes the decisions it does (Understandability 3).
16. ARS performs well under disturbed conditions (Competence 3).
17. ARS is very consistent (Predictability 3).
18. ARS will always make the same routing decision under the same circumstances (Reliability 2).
19. I trust ARS (Overall trust).

*Note.* ARS = Automated Route Setting.

### Study Design

**Figure 1.**
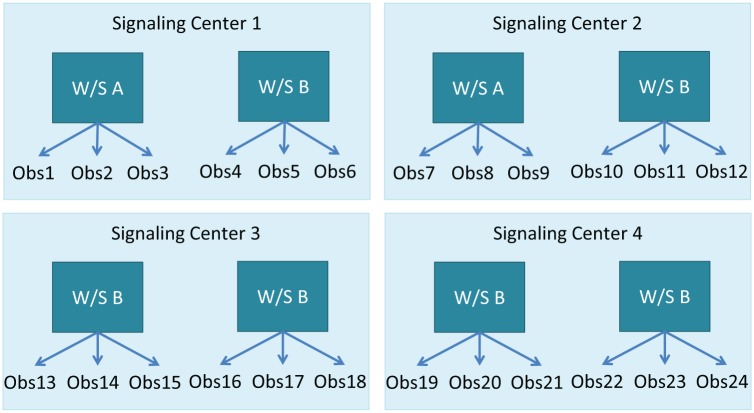
Study design.

Eight workstations in four signaling centers were included in the study. Workstations A in each signal box were comparable in terms of workload and the type of demands placed on the signalers. Workstations B were similarly chosen to be comparable to each other. The signaling centers chosen for the study were picked on the basis of the reported usage of ARS in each. Usage is reportedly low in Signaling Center 1, high in Signaling Center 2, and variable in Signaling Centers 3 and 4. Figure 1 describes the workstations involved in the study.

Complexity of workstations was measured using Operational Demand Evaluation Checklist (ODEC) scores and verified by subject matter experts. The ODEC tool was developed to measure the demand placed upon the signaler due to the infrastructure on a particular workstation (Pickup, Wilson, & Lowe, 2010). The tool measures quantifiable aspects of the workstation such as number of signals, number of level crossings, and speeds of trains and then ranks each entity as high, medium, or low. Visits were undertaken and data collected to complete ODECs for all workstations in the four signaling centers and, in order for the study to be comparable, the ODEC scores were matched as closely as possible for the workstations chosen for the study so the workstations were as similar as possible. In addition, subject matter experts were consulted to ensure that the specific demands of the chosen workstations were comparable; for example, the four workstations in Group A have high traffic levels through station areas whereas the four in Group B have a depot. Although every effort was made to make the workstations in the study as comparable as possible, there are no two areas on the railway that precisely match, and this variability must be accepted as a limitation of the study.

### Data Collection

Opportunity sampling was used for this study. Signalers were not specifically chosen for the study; the decision was based purely on who was working on the workstations of interest on the days planned for data collection and whether they were willing to take part in the study. On three occasions the same signaler was observed twice, but these repeated observations occurred by chance. Therefore, the total number of participants was 21 across 24 observations in total. All participants were male and had a minimum of 5 years’ signaling experience using ARS.

The observations were carried out at the same time of day on each workstation. The observation time was 4:30–6:00 p.m. The researcher arrived at the signaling center at approximately 4:00 p.m. and approached the signaler on the workstation of interest. Usually the signaler had been made aware of the study in advance to ensure he was happy with being observed, but in some cases this was not possible, and a brief outline of the study was required before proceeding. The study was then explained in more detail, and the signaler was given a consent form to read through and sign. Following this, the researcher asked about the current state of the area under the signaler’s control and whether there were any particular problems. Any instances of disruption or late-running trains were noted. Once everything was explained satisfactorily, the signaler was instructed to ignore the researcher as much as possible and to act as if she were not there. Once data collection commenced, signaler activity was coded every 5 s using the coding system outlined earlier.

At the end of the observation period, participants were given the questionnaire and asked to rate their agreement with each statement on a five-point Likert-type scale. They were also asked to give a short debrief on any unusual occurrences on the workstation during the observation. Finally, they were asked their most common reason for intervening during the observation and which intervention method they favored.

## Results

### Observation Results

**Figure 2.**
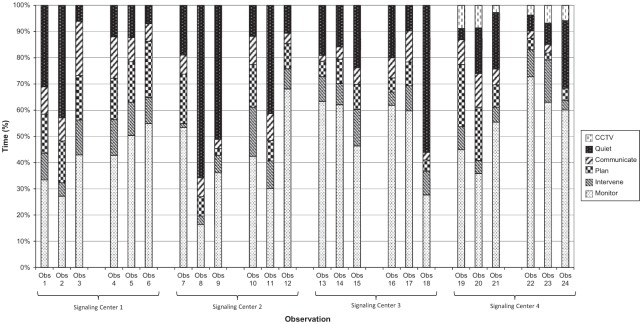
Observation results.

Figure 2 describes the results of the observations. Each bar on the graph describes the distribution of activity for one signaler, and each group of three bars describes the three observations for each workstation in the study. There are clear differences in activity between signalers. The same signaler was observed on Observation 3 and Observation 4, and these two graphs are remarkably similar. Observation 8 and Observation 11 also show the same signaler, but these graphs are different. In this case there was disruption during Observation 11 that contributed to the difference in the graphs. Finally, the same signaler was observed for Observation 23 and Observation 24, but these graphs show a big difference, particularly in intervention. It is not possible to account for this difference as the observations took place on the same workstation that was reportedly running smoothly on both days. The signaler on Observation 17 was the only one to choose not to use ARS.

Table 3 describes the average time dedicated to each behavior for each workstation in the study. The standard deviation also is given, and the high values for these illustrate the variability of the data on workstations. There is also considerable variation across control centers and workstations due to the differences in timetabled traffic and infrastructure in different geographical locations. This variability could not be controlled; however, the differences within workstations can be ascribed to individual differences as the traffic levels were identical for each observation.

**Table 3:** Average Percentage Occupancy and Standard Deviation per Workstation

	Monitoring	Intervention	Planning	Communications	Quiet Time	Closed-Circuit Television
Center 1 Workstation A	34 (8)	9 (4)	16 (1)	13 (7)	27 (19)	NA
Center 1 Workstation B	49 (6)	12 (2)	17 (3)	11 (5)	10 (3)	NA
Center 2 Workstation A	35 (19)	4 (3)	10 (8)	6 (2)	45 (24)	NA
Center 2 Workstation B	47 (19)	13 (6)	11 (4)	8 (4)	21 (17)	NA
Center 3 Workstation A	57 (10)	11 (3)	8 (2)	4 (2)	20 (4)	NA
Center 3 Workstation B	50 (19)	8 (3)	6 (3)	8 (5)	29 (24)	NA
Center 4 Workstation A	46 (10)	6 (2)	17 (7)	10 (4)	14 (9)	7 (3)
Center 4 Workstation B	65 (7)	10 (6)	4 (1)	2 (2)	13 (10)	6 (2)

*Note.* NA = not applicable.

The circumstances on the workstations during the observations were recorded. Out of the 24 observations, 13 had entirely smooth running with no problems whatsoever, 8 had minor problems that the signalers stated had little or no effect on their work, and 3 had more major infrastructure problems that had a slight effect on their work (Observations 1, 9, and 11). These 3 observations were examined, and the graph shows that the 3 observations with some disruption did not have the highest monitoring or intervention levels. It is likely that the disruption would have had some effect on the observed behavior of the signaler, but that effect was not large enough for these observations to be prominent.

### Monitoring

The mean percentage of time spent monitoring was 48%, a maximum of 73% and a minimum of 16% of the total 90-min observation. Two types of monitoring behavior were identified as a result of the pilot study and were coded during the remainder of the studies: active monitoring and passive monitoring. Passive monitoring was typically carried out for longer periods of time than active monitoring; the average length of time spent passively monitoring was 27 s, and the average length of time spent actively monitoring was 13 s. The proportion of passive monitoring was higher than that of active monitoring, with means of 27% and 21% of total time, respectively.

**Figure 3.**
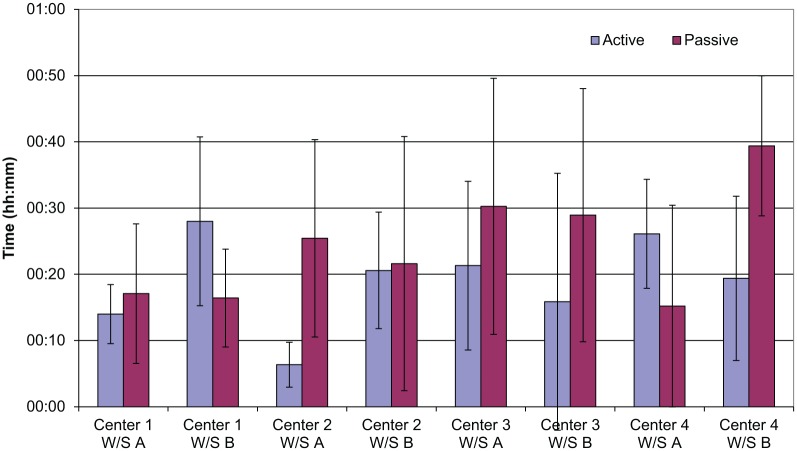
Monitoring results.

Figure 3 shows the average level of active and passive monitoring observed on each workstation in the study. The standard deviation is also shown, and the high standard deviation values suggest that the individual rather than the workstation drives the monitoring level. As the observations were carried out at the same time of day, the traffic encountered should have been very nearly identical. Although some of the workstations do show comparable monitoring levels (e.g., Observation 5 and Observation 6; see Figure 2), in light of the other data gathered it is likely that this is a coincidence.

### Intervention

The average percentage of time spent intervening during the course of an observation was 9%, the maximum was 19%, and the minimum was 1%. Two types of intervention were coded: use of the trackerball and use of the keyboard. The trackerball allows the signaler to set routes and other directive activities. These activities also can be achieved through the keyboard, but the keyboard also may be used to query ARS or to look up timetable information. Use of the trackerball was considerably higher than use of the keyboard. The average time for an intervention with the trackerball (8 s) was only slightly longer than that for keyboard interventions (6 s). Overall, use of the keyboard was very low compared with use of the trackerball, but use was highest in Signaling Center 1 (Observations 1–6).

**Figure 4.**
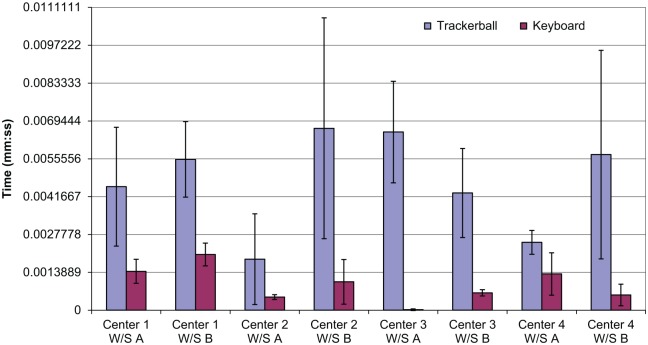
Intervention results.

Figure 4 shows the mean and standard deviation of trackerball and keyboard use for each workstation in the study. Similar to monitoring, intervention levels differed greatly between individuals, as can be seen by the high standard deviation for trackerball use. Since the three observations for each workstation were conducted at the same time of day, the train-running pattern should have been almost identical, and thus the workload and tasks encountered should have been very similar. An increase could be seen on workstations that experienced incidents during the course of the observation, but even then these were not the highest observed levels of intervention.

### Quiet Time

**Figure 5.**
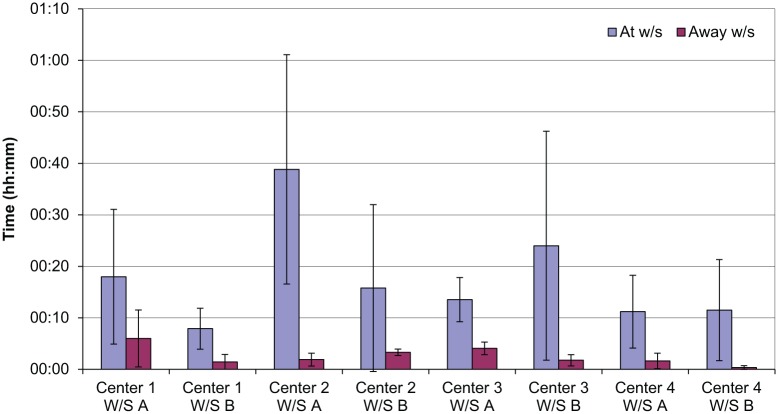
Quiet time results.

The average percentage of overall quiet time during the observations was 22%, with a maximum of 66% and a minimum of 4%. Two types of quiet time were coded: time spent at the workstation not actively involved in signaling and time spent away from the workstation. Individual quiet periods at the workstation lasted for an average of 20 s, compared with 1 min for periods away from the workstation. The longest quiet period spent at the desk without monitoring or engaging in any other signaling activity was 4 min, 35 s, but this was an unusually long time; the next highest time was 2 min, 55 s. As can be seen in Figure 5, quiet time at the workstation was considerably more common than quiet time away from the workstation. The longest time spent away from the workstation by any of the observed signalers was 2 min, 55 s.

### Other Activities

Automation was not expected to influence planning, communications, or CCTV activities during these observations. Communications are typically associated with disruption or failures, which did not occur during these observations. Planning is typically related to finding timetable information for approaching trains in a system unrelated to ARS, and CCTV is an entirely manual activity that has no relation to ARS. The results for these three activities are presented here briefly. The average percentage of total time spent occupied with planning activities was 11.3%, with a maximum of 23.2% and a minimum of 2.6%. Different centers tended to use different planning tools, but discussion of these differences is outside the scope of this study. The average percentage of time overall spent on communications was 7.8%, with a maximum of 20.7% and a minimum of 0.4%. The average time of an individual telephone call was 28 s, whereas conversations with other signalers on adjacent workstations were 9 s on average. CCTV screens required to operate level crossings were present only on the workstations in Signaling Center 4. Signalers were required to lower the barriers for each train and confirm that the crossing was clear for trains to pass. This occupied a reasonable chunk of the signalers’ time on these workstations, between 3% and 9% of the total observation time.

### Trust Results

**Figure 6.**
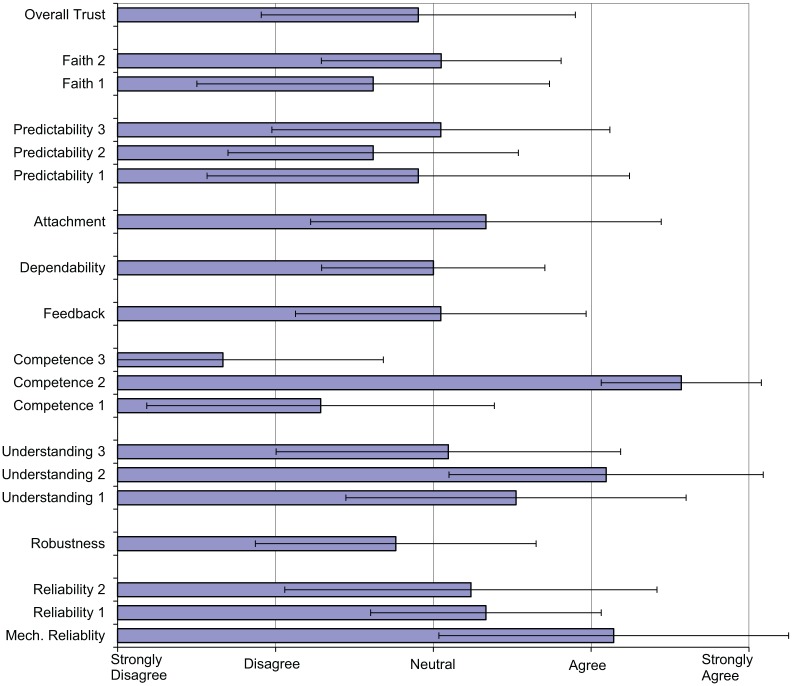
Trust questionnaire results.

The results of the questionnaires examining factors pertaining to each signaler’s perceived trust in the automation are described in this section. Although there were 24 observations, 3 signalers were observed twice but completed the questionnaire after only 1 observation. Therefore, there were 21 questionnaire respondents. The results of the questionnaire are shown in Figure 6.

### Statistical Analysis

In order to analyze further the results of the questionnaires, the signalers observed were divided into groups of high, medium, and low in terms of monitoring, intervention, and quiet time. In order to offset differences in activity driven by individual workstations, the groups were constructed by comparing the relative differences between the three signalers observed on each workstation. Planning, communications, and CCTV were not analyzed, as levels for these were likely to be affected by factors outside of the signalers’ direct control and not relevant to the central question of trust in automation. Table 4 shows the numbers of signalers assigned to the low, medium, and high groups for each of the three analyzed activities.

**Table 4:** Numbers of Signalers Assigned to Low, Medium, and High Groups

Group	Monitoring	Intervention	Quiet Time
Low	8	8	7
Medium	5	6	5
High	6	5	7

Each observation was compared to the other two observations on the same workstation to determine the groupings. Two observations (Observation 1 and Observation 9) were excluded from this part of the study because there was significant disruption on these workstations during the observations, and this may have affected the observed levels of each activity. These exclusions were in addition to Observation 3, Observation 11, and Observation 23, which were omitted as the signaler in each of these had already been observed and therefore had previously completed the trust questionnaire. The sample size was therefore 19. As data gathered using Likert-type scales can be regarded as pseudo-interval data (Tabachnick & Fidell, 2007), *t* tests were run between the high and low groups in each category to test for significant differences between the two groups, with higher levels of trust hypothesized for the low monitoring and intervention and high quiet time groups.

**Figure 7.**
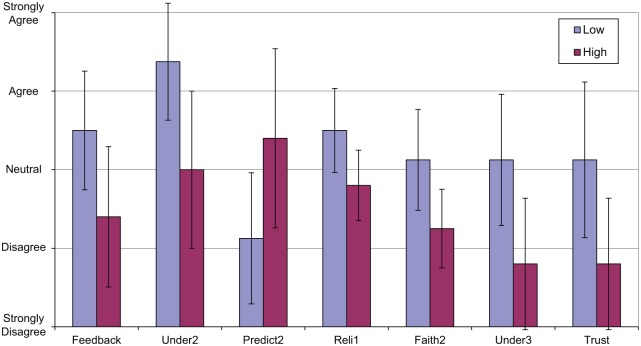
Significant differences between intervention groups.

No differences were found in terms of monitoring, and the null hypothesis was accepted for monitoring, but a number of differences were found in terms of intervention (see Figure 7):

- Feedback—“ARS gives explicit information on its intended actions,” *t*(11) = 2.385, *p* < .05. The low intervention group was more likely to agree with this statement.
- Understandability 2—“I understand how ARS works,” *t*(11) = 2.851, *p* < .05. The low intervention group was more likely to agree with this statement.
- Predictability 2—“ARS is very unpredictable; I never know what it is going to do,” *t*(11) = −2.337, *p* < .05. The high intervention group was more likely to agree with this statement.
- Reliability 1—“I can rely on ARS to function as it is supposed to,” *t*(11) = 2.434, *p* < .05. The low intervention group was more likely to agree with this statement.
- Faith 2—“Even if I have no reason to expect that ARS will be able to deal with a situation, I still feel certain that it will,” *t*(10) = 2.373, *p* < .05. The low intervention group was more likely to agree with this statement.
- Understandability 3—“I understand why ARS makes the decisions it does,” *t*(11) = 2.782, *p* < .05. The low intervention group was more likely to agree with this statement.
- Overall trust—“I trust ARS,” *t*(11) = 2.478, *p* < .05. The low intervention group was more likely to agree with this statement.

A difference was found between the groups for the overall understandability dimension, *t*(11) = 2.571, *p* < .05, with the low intervention group rating its understanding of the automation higher.

**Figure 8.**
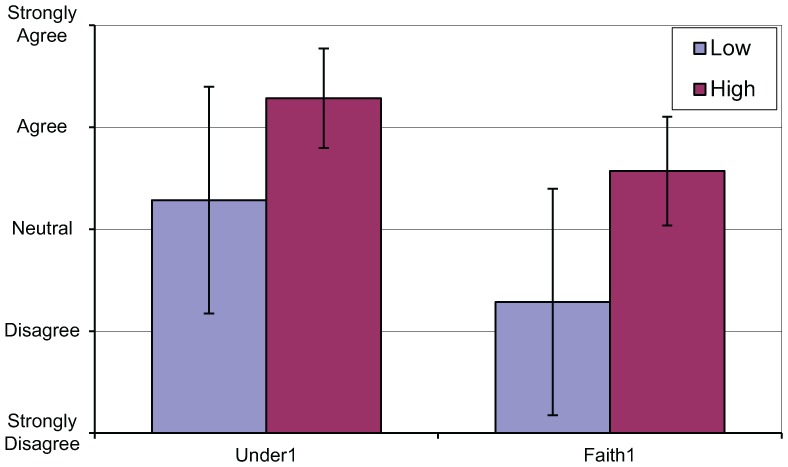
Significant differences between quiet time groups.

Two significant differences were found between signalers engaging in high and low levels of quiet time (see Figure 8):

- Understandability 1—“It is easy to understand what ARS does,” *t*(12) = −2.178, *p* < .05. Signalers displaying high levels of quiet time were more likely to agree with this statement.
- Faith 1—“If ARS makes a routing decision which I am uncertain about I have confidence that ARS is correct,” *t*(12) = −2.756, *p* < .05. Signalers displaying high levels of quiet time were more likely to agree with this statement.

## Discussion

### Observed Activities

Monitoring behavior showed considerable variation between observed signalers despite the observations on individual workstations experiencing similar conditions. It seems likely therefore that monitoring levels under normal running are driven by the individual rather than demand from the workstation.

Two types of monitoring were identified and coded during the study: active monitoring and passive monitoring. No reference to different states or levels of monitoring was found in the literature on automation. However, a similar concept arose in the discussion on differences between “active control” and “passive monitoring” (Endsley & Kiris, 1995), where it was suggested that automation induced passive processing of information that was inferior to active. Both active and passive monitoring were frequently engaged in throughout the study, but active monitoring was more common between interventions whereas passive monitoring was associated more with quiet time. It seems likely that rather than signalers’ working with automation constantly suffering from inferior information processing as is suggested in the literature, they actually actively process information when they believe decisions may be required and engage in a more relaxed form of monitoring (passive monitoring) when they feel the demands of the workstation are lesser. Although it was not possible to determine how much attention the signaler paid during passive monitoring, there are frequent examples of interventions following a period of passive monitoring, so it can be concluded that information still is being processed. Cowan (1988) suggests that attention can be automatically triggered even during passive information-processing states. If this is the case, the use of different levels of monitoring behavior may be a very effective strategy for reducing workload associated with monitoring, which has been shown to be high (Warm, Dember, & Hancock, 1996), while still maintaining awareness of the system.

High levels of monitoring (either active or passive) were associated with low levels of quiet time, and low monitoring was associated with high levels of quiet time. This would seem to suggest that monitoring and quiet time are interchangeable and that signalers who can find a distraction (i.e., someone to talk to in most cases) will use some of the time otherwise used for monitoring purposes. There is probably a lower threshold of monitoring below which the signalers would feel uncomfortable, but further research would be required to identify what this might be. However, establishing that boundary would contribute to understanding the necessary levels of operator awareness of the system. The association of active monitoring with interventions and passive monitoring with quiet time provides some validation of the decision to differentiate between the two types of monitoring. More research is required to look at monitoring behaviors alone to identify what triggers each one and the quantity, quality, and type of information gathered at each level. It is important to note that all observed signalers engaged in routine monitoring behavior, and the longest observed period when they were away from the workstation and could not monitor it was just less than 3 min. It would appear that monitoring is a critical ongoing activity for the signalers that they are not willing to neglect. This suggests that they place a high priority on maintaining awareness of their control area, even when ARS is running all trains.

The results show a high degree of variation in intervention levels between signalers. Unfortunately, it is not obvious from the data why some observations had higher intervention levels than others. It is clear that it is not totally due to particular circumstances on the workstation, as the events experienced should have been very similar. This is a limitation of the observational method, which documents what happens but not why. For example, perhaps some signalers intervened in advance and were thus able to deal with problems quite quickly, perhaps even making some interventions to prevent a situation’s developing, or some interventions may be more efficient than others. Therefore, like monitoring levels, intervention levels appear to be driven by the individual rather than the workstation, and research supports the theory that individual differences may account for these differences (Merritt & Ilgen, 2008).

### Trust

Overall reported trust (Question 19) in the ARS system was neutral, with three strong disagreements. Reliability was found to be relatively high, particularly in terms of mechanical reliability or availability. The results were more varied for the related questions about competence, with participants’ universally agreeing that ARS performs well under normal running conditions but strongly disagreeing that it works well under disrupted (abnormal) conditions. The question about robustness, to which the responses were primarily negative, reinforces this view. The majority of participants also indicated that they considered ARS less competent than themselves. This indicates both the confidence they have in their own abilities and their lack of confidence in the automation. It is interesting to note that their lack of confidence in ARS is not sufficient to fully disable the system, and this suggests that they view it as a useful aid but not one with the same capabilities as themselves. The impact of this relative lack of competence of the automation, particularly in degraded modes, on trust may not be very great; signalers may simply calibrate their trust accordingly by developing a set of situations under which they trust ARS and assuming manual control or inhibiting the automation for other situations. The impact on workload is likely to be greater, with the automation offering the least support when the demands are greatest. The responses to the questions about understanding were largely positive, but the final question about why ARS makes the decisions it does scored lowest. This indicates that signalers may have a lesser understanding of why ARS makes certain decisions than of what it is doing and how it does it. The responses about predictability were more negative, and it appears that individuals’ perceived ability to predict the automation varies widely.

The results indicate that correlations can be found between the observed behaviors of signalers and their reported trust in ARS, particularly in relation to the amount of time spent intervening. Although the sample size was small, the direction of the differences between groups all indicate that lower trust results in higher intervention, and the significant difference found in reported levels of overall trust and the high and low intervention groups provides some confidence that the methodology can distinguish between participants with varying levels of trust in the ARS system.

Differences were found in questions relating to feedback, understanding, predictability, reliability, and faith. Table 5 summarizes the dimensions on which significant differences were found between groups of high and low observed monitoring, intervention, and quiet time. Apart from research on reliability and feedback (Dzindolet et al., 2003; Wiegmann et al., 2001), no research is known to have found empirical evidence supporting the relationship of understanding, predictability, and faith to automation usage. Although the importance of developing understanding and accurate mental models and designing transparent automation (Chen et al., 2014; Lyons, Koltai, et al., 2016) is stated in much of the literature, the empirical research has focused largely on the reliability and competence dimensions of trust and suggests that these are the most critical in influencing automation usage. However, only one question out of six on these aspects (“I can rely on ARS to function as it is supposed to”) showed a difference between high and low levels of intervention, and no difference was found in terms of the competence question. In real-world industrial systems, operator competence is developed through rigorous training and maintained through competence-management systems, and this investment may negate the role of competence found in laboratory studies.

**Table 5:** Summary of Significant Differences Between Groups

	Monitoring	Intervention	Quiet Time
Reliability		*	
Robustness			
Understanding		3*	*
Competence			
Feedback		*	
Dependability			
Attachment			
Predictability		*	
Faith		*	*
Overall trust		*	

Note. *indicates the number of significant differences found.

In this study, other dimensions of trust were more prominent. In particular, the ability to understand the automation showed significant differences in two of the three questions as well as across the group (i.e., the three significant differences indicated in Table 5). Feedback and predictability also showed significant differences between signalers exhibiting high and low levels of intervention. The results from this study suggest that for application in real-world systems, future research should focus on the role of understandability in terms of both how it influences trust and automation usage and how it can be developed and supported. Safety-critical systems are always likely to be reliable, at least in a mechanical sense, whereas their competence is often dictated by the maturity of the technology and the constraints of the system being controlled. Feedback/transparency and understanding, leading to a greater ability to predict the automation, can both be facilitated through a variety of methods, including improved function allocation, innovative human-machine interface design, and thorough training. As automation increases in scope and complexity, the role of understanding is likely to become more critical.

The significant differences relating to understanding and faith between signalers exhibiting high and low levels of quiet time suggest that operators with a higher level of understanding and faith in the automation are more content to allow the automation to continue unmonitored as well as intervene less often. This may have important implications for operator complacency, particularly in systems where there is a safety-critical element to monitoring such as automated driving.

## Conclusions

This study aimed to develop a method to investigate expert operator use of and trust in an advanced, real-world automated system under normal operational conditions. The combination of the observation method and questionnaire proved effective for addressing this aim and identifying differences between participants, particularly in terms of levels of intervention.

The study only partially replicated the findings from the lab-based research documented in the literature, with understanding of the automation rather than reliability or competence emerging as the main differentiator between high and low levels of intervention among participants. On the basis of the results, we propose that understanding and feedback may be more relevant for real-world safety-critical systems than reliability or competence and that operator trust in real-world automation may be fundamentally distinct from trust in laboratory automation. This has strong implications for future research on human interaction with automation systems and on the design of future automated systems, with the necessity of a renewed focus on improving automation feedback and greater investigation into the role and development of mental models in expert operators. This is particularly important in the development of new automation concepts, for example, in the automotive domains, and in extension of automation concepts, for example, through the Internet of things. However, the study was based in a single domain with a single automated system. Application of this methodology in other well-established, highly automated domains is necessary to determine how generalizable these results may be and to explore whether different domains and automated systems have different requirements.

This study also introduced the idea of different levels of monitoring behavior and proposed that these may be an effective way for expert operators to regulate workload and effort. Further research into this topic, for example, using eye-tracking and other physiological data collection, would be extremely beneficial in supporting operator interaction with the automated systems of the future.

## Key Points

- An observation methodology was developed and combined with a survey method to investigate trust in real-world automation use.
- Different types of monitoring were observed, and it is suggested that these may be an effective way for expert operators to regulate workload and effort.
- Understanding of the automation rather than reliability or competence emerged as the main differentiator between high and low levels of intervention among participants in this study.
- Understanding and feedback may be more relevant for real-world, safety-critical systems than reliability or competence, and operator trust in real-world automation may be fundamentally distinct from trust in laboratory automation.
